# Coronary Artery Bypass Surgery in Patients on Dialysis: In-Hospital Outcomes from UK Registry Analysis

**DOI:** 10.1093/icvts/ivaf291

**Published:** 2025-12-03

**Authors:** Muhammed A Mashat, Tim Dong, Rahul Kota, Ettorino Di Tommaso, Pradeep Narayan, Charles Tan, Cha Rajakaruna, Eltayeb Mohamed Ahmed, Gianni D Angelini, Daniel P Fudulu

**Affiliations:** Bristol Heart Institute, University of Bristol, Bristol, BS2 8ED, UK; King Abdulaziz University, Jeddah, 21589, Saudi Arabia; Bristol Heart Institute, University of Bristol, Bristol, BS2 8ED, UK; Bristol Heart Institute, University of Bristol, Bristol, BS2 8ED, UK; Bristol Heart Institute, University of Bristol, Bristol, BS2 8ED, UK; Bristol Heart Institute, University of Bristol, Bristol, BS2 8ED, UK; Department of Cardiac Surgery, Rabindranath Tagore International Institute of Cardiac Sciences, Narayana Health, Kolkata, 700099, India; Bristol Heart Institute, University of Bristol, Bristol, BS2 8ED, UK; Bristol Heart Institute, University of Bristol, Bristol, BS2 8ED, UK; Bristol Heart Institute, University of Bristol, Bristol, BS2 8ED, UK; Bristol Heart Institute, University of Bristol, Bristol, BS2 8ED, UK; Bristol Heart Institute, University of Bristol, Bristol, BS2 8ED, UK

**Keywords:** coronary artery bypass graft, CABG, dialysis, CKD, CAD

## Abstract

**Objectives:**

Chronic kidney disease requiring dialysis significantly increases the risks of coronary artery disease. However, there is limited data on this high-risk patient population requiring coronary artery bypass grafting. Using a UK national registry, we investigated the impact of preoperative dialysis on in-hospital mortality and early morbidity in patients undergoing coronary artery bypass graft (CABG).

**Methods:**

A retrospective analysis of National Adult Cardiac Surgery Audit data between January 1996, 2, and March 31, 2019, identified patients who underwent first-time isolated CABG. Propensity matching was performed to balance the baseline characteristics between dialysis and non-dialysis patients, yielding 633 matched pairs. We evaluated trends in CABG among dialysis patients and EuroSCORE 2 performance in predicting in-hospital mortality (calibration, discrimination, and clinical utility).

**Results:**

There was a steep increase in CABG operations in dialysis patients after 2011. EuroSCORE 2 showed poor calibration, discrimination, and minimal clinical benefit in predicting mortality in dialysis cases. Dialysis patients exhibited a significantly higher in-hospital mortality rate (7.9% vs 2.1%, *P* < .001) than non-dialysis patients. The dialysis patients had longer median hospital stays (12 vs 9 days, *P* < .001) and a higher rate of return to the theatre for bleeding (5.5% vs 2.7%, *P* = .034). We found no difference in postoperative neurological deficit rates between the 2 cohorts. The odds ratio of in-hospital mortality for the dialysis vs non-dialysis patients was 4.62, *P* < .001, 95% (CI: 2.54-8.4). Significant predictors of mortality in the dialysis CABG cohort included advanced age (OR: 2.48), New York Heart Association class IV (OR: 3.06), and pulmonary hypertension (OR: 11.91).

**Conclusions:**

There has been an overall increase in coronary artery bypass operations performed in renal dialysis-dependent patients in the UK. Preoperative chronic dialysis is associated with considerable in-hospital mortality, return to theatre for bleeding and prolonged hospital stay. EuroSCORE 2 has poor predictive performance in this patient cohort.

## Graphical abstract

**Figure:**
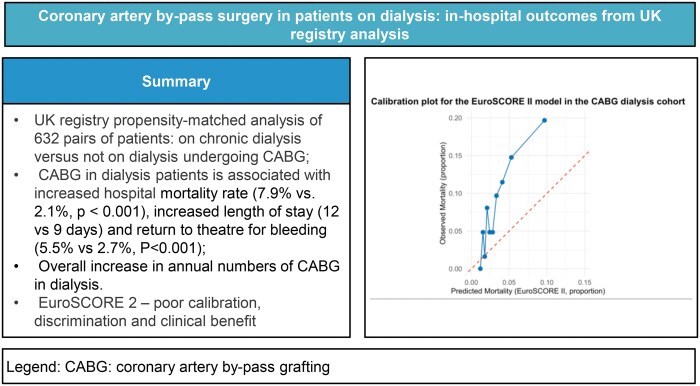

## INTRODUCTION

Coronary artery disease (CAD) is a leading cause of morbidity and mortality worldwide,^1^ particularly among patients with chronic kidney disease (CKD).^2^ CKD provokes a systemic and a chronic proinflammatory state that accelerates vascular and myocardial remodelling processes. This chronic process contributes to atherosclerotic lesions and vascular calcification, mirroring expedited cardiovascular ageing.^2^ The prevalence of renal dialysis for end-stage kidney disease has increased throughout the years, reaching 1%-1.5% of the UK population in 2021.^1^^,^^3^ Multivessel and diffuse coronary lesions with rigid calcified vessels often characterize the increased incidence of CAD in dialysis patients. The histological prevalence of vascular calcifications is increased more than 40-fold in patients with CKD compared to patients without CKD.^2^^,^^4^ All these factors pose unique technical challenges to percutaneous coronary intervention (PCI) due to complications such as stent under-expansion and subsequent restenosis or thrombosis.^4–7^

Coronary artery bypass grafting in patients undergoing renal dialysis is an alternative revascularization strategy to PCI, shown to be associated with repeat revascularization rates.^8^ However, it comes at the cost of substantial mortality.^9^^,^^10^ This study aims to fill the knowledge gaps outlined above by investigating the trends, in-hospital outcomes and predictors in patients on chronic preoperative dialysis undergoing CABG in a large UK national registry cohort.

## METHODS

### Study design and setting

We retrospectively analysed data collected from the National Adult Cardiac Surgery Audit (NACSA), obtained from the National Institute for Cardiovascular Outcomes Research (NICOR) central cardiac database. The NACSA registry prospectively collects demographic and pre-, peri-, and postoperative clinical data for cardiac surgery procedures performed in the UK, aiming to benchmark surgical practice. The flow of data and quality assurance processes has been described in our previous work.^11^^,^^12^ The Health Research Authority and Health and Care Research Wales approved the study in 2020, IRAS project ID 257758, and a waiver for patients’ consent was obtained. This study was conducted in accordance with the Declaration of Helsinki.

### Patients

**Figure 1.**
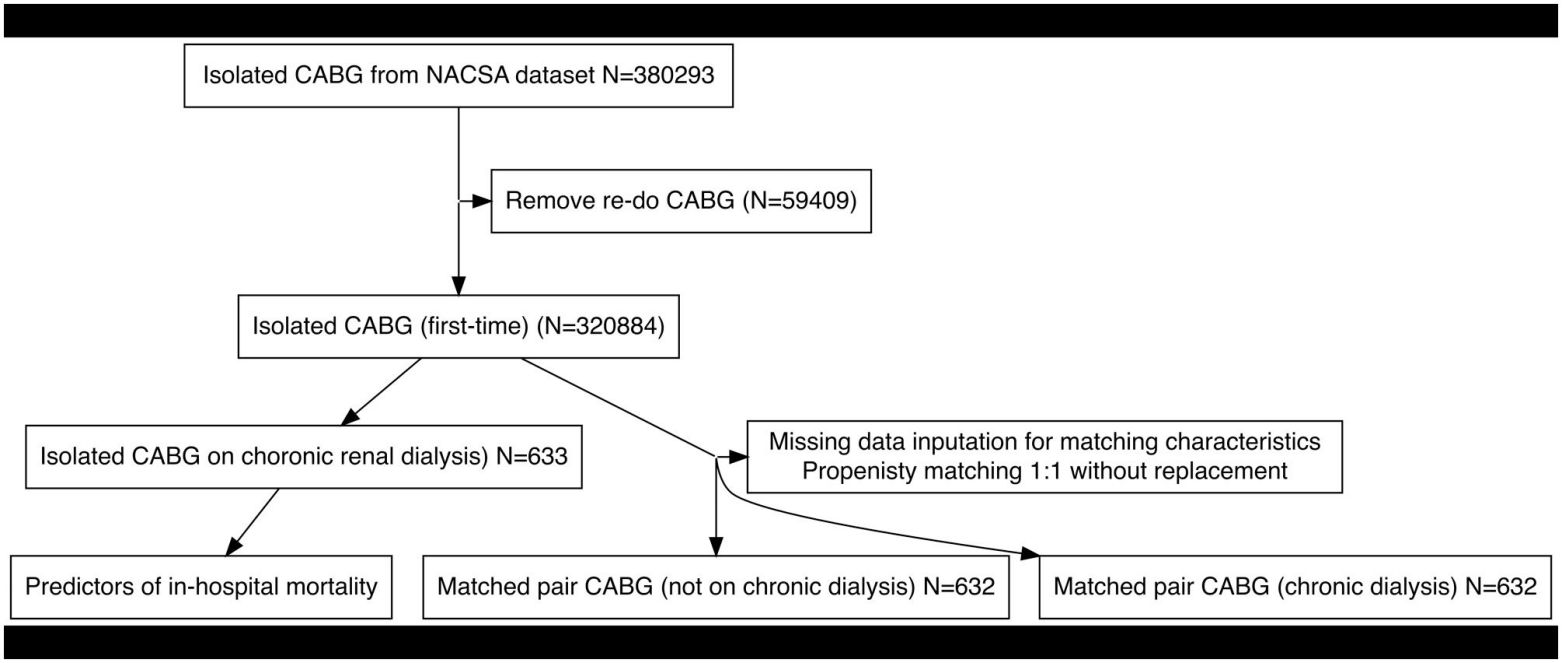
Data Flow Diagram. Abbreviations: CABG, coronary artery bypass graft; NACSA, National Adult Cardiac Surgery Audit

Patients who underwent first-time isolated CABG and were on preoperative chronic dialysis (>6 weeks preoperatively) were included and compared against a propensity-matched cohort of patients not on dialysis. These procedures were performed between 2008 and 2019. The data flow diagram is displayed in **Figure 1**.

### Outcomes

The primary outcome was in-hospital mortality. Secondary outcomes included neurological injury as the composite outcome of reversible neurological deficit (transient ischaemic attack [TIA]) and permanent neurological deficit (cerebrovascular accident [CVA]), return to theatre for bleeding or cardiac tamponade, and hospital length of stay, and predictive performance of the EuroSCORE 2 model in dialysis patients.

### Statistical methods

#### Baseline characteristics analysis (unmatched data)

Categorical variables were summarized as counts and percentages and compared using Pearson’s chi-squared test or Fisher’s exact test. We used the Shapiro-Wilks test to assess the normality of the distribution of continuous data. Our continuous data were non-normally distributed, so they were summarized as medians with IQRs and analysed using the Wilcoxon rank-sum test. Missing data in the matching baseline characteristics were treated with missing data imputation (“mice”, R package). We did not input missing data in the outcome variables. Missingness was present in the following matching variables: number of grafts (0.95%), whether the case was performed on- or off-pump (1.83%), and body mass index (BMI) (5.54%).

#### Propensity-matched analysis

Because chronic dialysis patients are a very comorbid population, we undertook a propensity-matching approach to understand the effect of dialysis alone on outcomes. Propensity score matching was performed using 1:1 nearest neighbour matching (matching caliper 0.2) without replacement using the following baseline characteristics from the NACSA dataset that we decided are best related to the outcome. Matching variables are depicted in **Table 1**.

**Table 1.** Baseline Characteristics and Outcomes between Dialysis and Non-Dialysis Patients Undergoing CABG before Matching

Characteristic	Before matching				After matching			
	**No dialysis** ***N* = 320 251^a^**	**Dialysis** ***N* = 633^a^**	Difference^b^	95% CI^b^	**No dialysis** ***N* = 632^a^**	**Dialysis** ***N* = 632^a^**	Difference^b^	95% CI^b^
**Gender**	61 380 (19%)	127 (20%)	−0.02	−0.10 to 0.06	138 (22%)	127 (20%)	0.04	−0.07 to 0.15
**Age (years)**	67 (59, 73)	64 (56, 71)	0.25	0.17 to 0.33	64 (56, 72)	64 (56, 71)	0.04	−0.07 to 0.15
**BMI**	27.9 (25.3, 31.0)	28.0 (24.4, 31.5)	0.01	−0.07 to 0.08	28.1 (25.5, 31.3)	28.0 (24.4, 31.6)	0.03	−0.08 to 0.14
**Neurological dysfunction**	6312 (2.0%)	32 (5.1%)	−0.17	−0.25 to −0.09	25 (4.0%)	32 (5.1%)	−0.05	−0.16 to 0.06
**Recent MI**	76 004 (24%)	262 (41%)	−0.38	−0.46 to −0.31	270 (43%)	261 (41%)	0.03	−0.08 to 0.14
**Pulmonary disease**	36 551 (11%)	87 (14%)	−0.07	−0.15 to 0.01	97 (15%)	86 (14%)	0.05	−0.06 to 0.16
**CCS 4**	45 858 (14%)	89 (14%)	0.01	−0.07 to 0.09	74 (12%)	88 (14%)	−0.07	−0.18 to 0.04
**NYHA IV**	10 368 (3.2%)	37 (5.8%)	−0.13	−0.20 to −0.05	33 (5.2%)	36 (5.7%)	−0.02	−0.13 to 0.09
**Pulmonary hypertension**	1020 (0.3%)	5 (0.8%)	0.06	−0.01 to 0.14	6 (0.9%)	5 (0.8%)	0.02	−0.09 to 0.13
**Diabetes on insulin**	23 473 (7.3%)	248 (39%)	−0.81	−0.89 to −0.74	244 (39%)	247 (39%)	−0.01	−0.12 to 0.10
**Poor LV function**	93 904 (29%)	237 (37%)	−0.17	−0.25 to −0.09	242 (38%)	237 (38%)	0.02	−0.09 to 0.13
**Moderate LV function**	36 911 (12%)	136 (21%)	−0.27	−0.35 to −0.19	139 (22%)	135 (21%)	0.02	−0.09 to 0.13
**PVD**	41 082 (13%)	168 (27%)	−0.35	−0.43 to −0.27	161 (25%)	167 (26%)	−0.02	−0.13 to 0.09
**Preop atrial fibrillation**	10 547 (3.3%)	37 (5.8%)	−0.12	−0.20 to −0.04	37 (5.9%)	37 (5.9%)	0.00	−0.11 to 0.11
**Emergency case**	6846 (2.1%)	21 (3.3%)	−0.07	−0.15 to 0.01	13 (2.1%)	20 (3.2%)	−0.07	−0.18 to 0.04
**Urgent case**	103 178 (32%)	303 (48%)	−0.32	−0.40 to −0.25	309 (49%)	303 (48%)	0.02	−0.09 to 0.13
**Pump case**	271 072 (85%)	513 (81%)	0.10	0.02 to 0.17	495 (78%)	513 (81%)	−0.07	−0.18 to 0.04
**Number of grafts**			0.06	−0.01 to 0.14			−0.05	−0.16 to 0.06
1	16 002 (5.0%)	40 (6.3%)			49 (7.8%)	40 (6.3%)		
2	68 751 (21%)	140 (22%)			127 (20%)	140 (22%)		
3	153 505 (48%)	336 (53%)			312 (49%)	336 (53%)		
4	67 746 (21%)	101 (16%)			123 (19%)	101 (16%)		
5	9155 (2.9%)	15 (2.4%)			9 (1.4%)	14 (2.2%)		
6	840 (0.3%)	1 (0.2%)			1 (0.2%)	1 (0.2%)		
**One arterial graft**	240 636 (75%)	520 (82%)			520 (82%)	519 (82%)	0.00	−0.11 to 0.11
**Two arterial graft or more**	45 936 (14%)	9 (1.4%)	0.49	0.42 to 0.57	8 (1.3%)	9 (1.4%)	0.01	−0.10 to 0.12
**Unmatched variables**								
Preop creatinine (μmol/L)	85 (74, 99)	417 (175, 597)	−1.8	−1.9 to −1.7	86 (74, 104)	417 (175, 596)	−1.7	−1.9 to −1.6
Aortic cross-clamp time(min)	43 (30, 57)	45 (30, 63)	−0.07	−0.15, 0.01	41 (27, 58)	45 (30, 63)	−0.15	−0.27 to −0.03
Unknown	41 745	62			109	62		

Abbreviations: BMI, Body mass index; CCS 4, Canadian Cardiovascular Society class 4; LV, left ventricular; NYHA IV, New York Heart Association class IV; PVD, Peripheral Vascular Disease.

a *n* (%); median (Q1, Q3).

b Standardized mean difference.

After matching, all standardized mean differences (SMD) for the covariates were checked using love plots, and the adequate balance was set to be below 0.1 (**Figure 1**). All SMDs were below 0.1. Matched variables were compared using a paired *t*-test for continuous data and the McNemar test for binary data.^13^ Assessment of covariate overlap is provided in the **Figure S1**.

#### Estimating the effect of chronic dialysis in the matched cohort

To estimate the effect of preoperative chronic dialysis versus no dialysis, we fitted a logistic regression model with mortality as a binary outcome and the same variables used in matching (double adjustment). We included the full matching weights in the estimation. The “glm ()” function was used to fit the outcome. We used the “marginal effects” package to perform g-computation in the matched sample to estimate the average treatment effect in patients on preoperative chronic dialysis undergoing CABG compared to non-dialysis cases. As previously reported, a cluster-robust inference with matching stratum membership as the clustering variable was used to estimate the marginal odds ratio (OR), relative risk (RR) and risk difference (RD) for in-hospital mortality in the matched population.^14^^,^^15^

#### Predictors of mortality in the dialysis cohort only

We further investigated the predictors of adverse mortality in the patients on preoperative chronic dialysis only by constructing a logistic regression model (“glm”). The initial model contained the following variables: age (numerical variable), female sex (Yes/No), preoperative atrial fibrillation (AF) (Yes/No), preoperative neurological dysfunction (Yes/No), on- or off-pump case (Yes/No), hypertension (Yes/No), BMI (numeric variable) (Yes/No), recent myocardial infarction (MI) (Yes/No), pulmonary disease (Yes/No), New York Heart Association (NYHA) IV (Yes/No), Canadian Cardiovascular Society (CCS) 4 (Yes/No), diabetes on insulin (Yes/No), poor ejection fraction (<30%) (Yes/No), moderate ejection fraction (31%-50%) (Yes/No), peripheral vascular disease (Yes/No), emergency surgery (Yes/No), urgent surgery (Yes/No), one arterial graft (Yes/No), 2 or more arterial grafts (Yes/No), number of grafts (numerical variable) (Yes/No). To avoid overfitting the model and account for the potential low event per variable ratio in the sample, we have used a backward selection process using the “MASS” R package, and from the initial model with an Akaike information criterion (AIC) of 319.57, we arrived at an optimal model with 4 predictors with an AIC of 302.06 (**Table 3**).

**Table 2.** Baseline Characteristics and Outcomes between Dialysis and Non-Dialysis Patients Undergoing CABG after Matching

Baseline characteristics post matching			
Characteristic	Conduit strategy		***P*-value** ^b^
	**No dialysis** *N* = 632^a^	**Dialysis** *N* = 632^a^	
**In-hospital mortality**	13 (2.1%)	49 (7.9%)	<.001
Missing	3	15	
**Postoperative CVA**	6 (0.9%)	5 (0.8%)	>.9
**Postoperative TIA**	2 (0.3%)	5 (0.8%)	.4
**Return to theatre for bleeding or tamponade**	17 (2.7%)	35 (5.5%)	.014
**Total length of stay (days)**	9 (7, 14) days	12 (8, 23) days	<.001
Missing	16	6	

Abbreviations: CVA, cerebrovascular accident; TIA, transient ischaemic attack.

a Median (IQR) or frequency (%).

b McNemar’s chi-squared test with continuity correction; paired *t*-test; random intercept logistic regression; McNemar’s chi-squared test.

**Table 3.** Predictors of In-Hospital Mortality in the Dialysis Cohort Retained in the Final Model following Stepwise Backward Regression

	Mortality		
Predictors	Odds ratios	95% CI	*P*-value
Advanced age	1.05	1.01-1.08	.008
NYHA IV	3.72	1.34-9.33	.007
Short cross-clamp time	0.99	0.97-1.00	.025
Emergency surgery	2.83	0.74-8.79	.092
Observations	558		
*R* ^2^ Tjur	0.045		

Abbreviations: NYHA, New York Heart Association.

#### EuroSCORE 2 prediction performance in the CABG dialysis cohort

Model calibration was assessed by comparing the predicted and observed mortality proportions for the EuroSCORE II model in the CABG dialysis cohort. Predicted mortality was expressed as the mean expected probability from the EuroSCORE II model, and observed mortality was calculated as the proportion of deaths within each risk group.

A calibration plot was constructed in R using the *ggplot2* package, plotting observed mortality on the *y*-axis against predicted mortality on the *x*-axis.

Model discrimination was assessed using the receiver operating characteristic (ROC) curve. The area under the curve (AUC) with 95% CIs was calculated using the *pROC* package in R. An AUC of 1.0 indicates perfect discrimination. In contrast, an AUC of 0.5 represents no better performance than chance. The clinical utility of the model was assessed using decision-curve analysis (DCA) implemented via the *rmda* package in R.

Net benefit was calculated for the EuroSCORE II model across a range of threshold probabilities and compared with “treat all” (assuming all patients are high risk) and “treat none” (assuming no patients are high risk).

## RESULTS

### Trends in annual volume of cases and observed versus expected mortality

**Figure 2.**
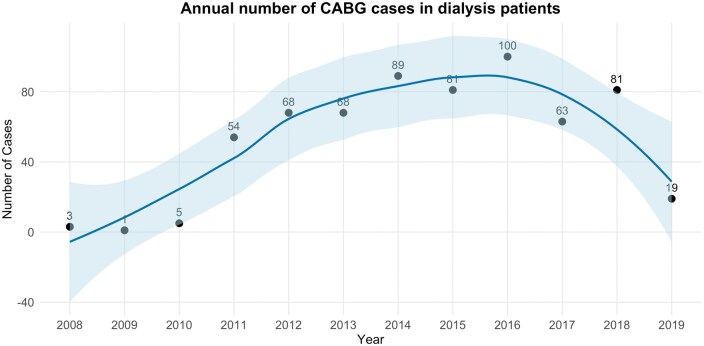
Number of Coronary Artery Bypass Graft (CABG) Cases per Year in the Dialysis-Matched Cohort. The individual data points represent annual case numbers, while the solid line depicts a locally weighted regression (LOESS) curve that shows temporal trends. The shaded area represents the 95% CI for the smoothed estimate

We noted a steep increase in the volume of CABG performed in dialysis cases from 2011 onwards, with over 50 cases per year and a peak of 100 cases in 2016 (**Figure 2**) and after this, a downward trend in cases.

### Unmatched population

**Figure 3.**
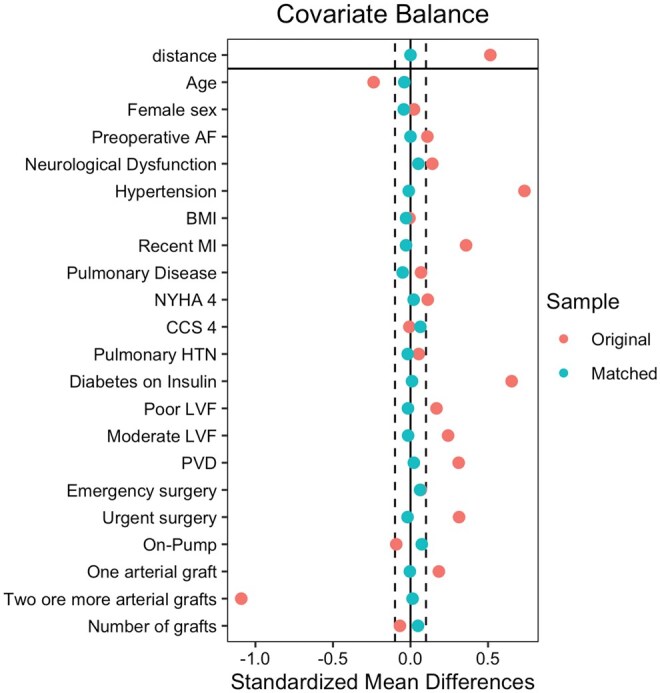
Love Plot Depicting the Quality of Matching between Dialysis Cases and Non-Dialysis Cases. Abbreviations: AF, atrial fibrillation; BMI, body mass index; CCS, Canadian Cardiovascular Society; HTN, hypertension; LVF, Left Ventricular Function; MI, myocardial infarction; NYHA, New York Heart Association; PVD, peripheral vascular disease

Three hundred eighty thousand two hundred ninety-three patients underwent first-time isolated CABG procedures from the NACSA database between January 2, 1996, and March 31, 2019. A total of 59 409 patients were excluded after applying the exclusion criteria (redo procedures). A total of 320 894 patients were included in the propensity-matched analysis (**Figure 1**): 632 matched pairs. **Table 1** illustrates the balance in the baseline characteristics of both groups before and after propensity matching. **Figure 3** depicts the quality of matching, ensuring SMDs below 0.1.

### Outcomes after matching

Patients on preoperative dialysis had a significantly higher in-hospital mortality (7.9% vs 2.0%, *P* < .001) (**Table 2**). Furthermore, the median length of stay was longer for dialysis patients than for non-dialysis patients (12 days vs 9 days, *P* < .001). Patients on preoperative dialysis were also more likely to return to theatre for bleeding or tamponade (5.5% vs 2.7%, *P* = .014). The occurrence of transient ischaemic attacks was comparable between patients undergoing preoperative dialysis (0.5%) and those on non-dialysis (0.3%) (*P* = .4), as was the incidence of CVA (0.8% vs 0.9%, *P* > .9).

After adjustment using cluster-robust inference in the matched sample, the marginal OR of mortality in dialysis patients compared to non-dialysis cases was 4.62, *P* < .001, 95% CI 2.54-8.4; the marginal RR was 4.33 and 95% CI 2.42-7.75; and the absolute RD was 0.061, 95% CI 0.03-0.08.

### Predictors of in-hospital mortality in the dialysis cohort

In investigating the predictors of in-hospital mortality among patients on dialysis who underwent CABG, several predictors were retained in the final model after stepwise backward regression analysis (**Table 3**). Old age was associated with an OR of 1.05 in-hospital mortality (95% CI: 1.01-1.08, *P* = .008). Furthermore, patients with NYHA class IV symptoms had a 3-fold increase in the risk of in-hospital death (OR: 3.72, 95% CI: 1.34-9.33, *P* = .025). While emergency surgery appeared to elevate the risk (OR: 2.83), this association did not reach the statistical significance threshold (95% CI: 0.74-8.79, *P* = .09).

### Performance of the EuroSCORE 2 model in the prediction of mortality in patients on dialysis undergoing CABG

#### Calibration

**Figure 4.**
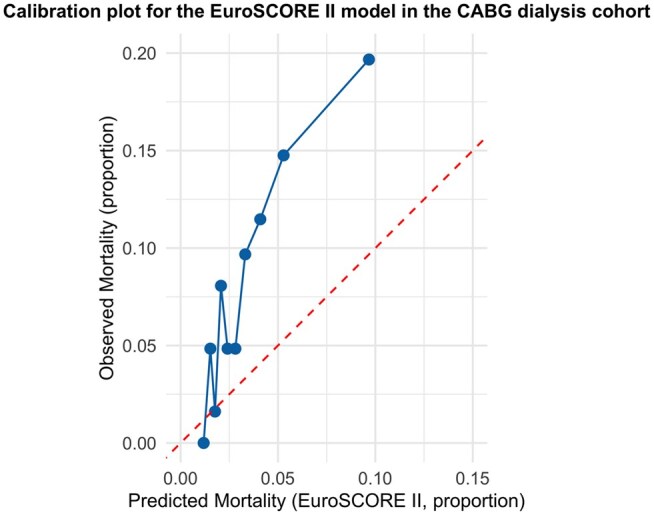
Calibration Plot for the EuroSCORE 2 Model in the Dialysis Cohort. Abbreviations: CABG, coronary artery bypass graft

The calibration plot (**Figure 4**) shows that the EuroSCORE II model underpredicts mortality in the dialysis cohort. The calibration slope was 0.02, and the intercept was 0.004. The calibration slope indicates no correlation between predicted and observed outcomes, suggesting very poor calibration.

#### Discrimination

**Figure 5.**
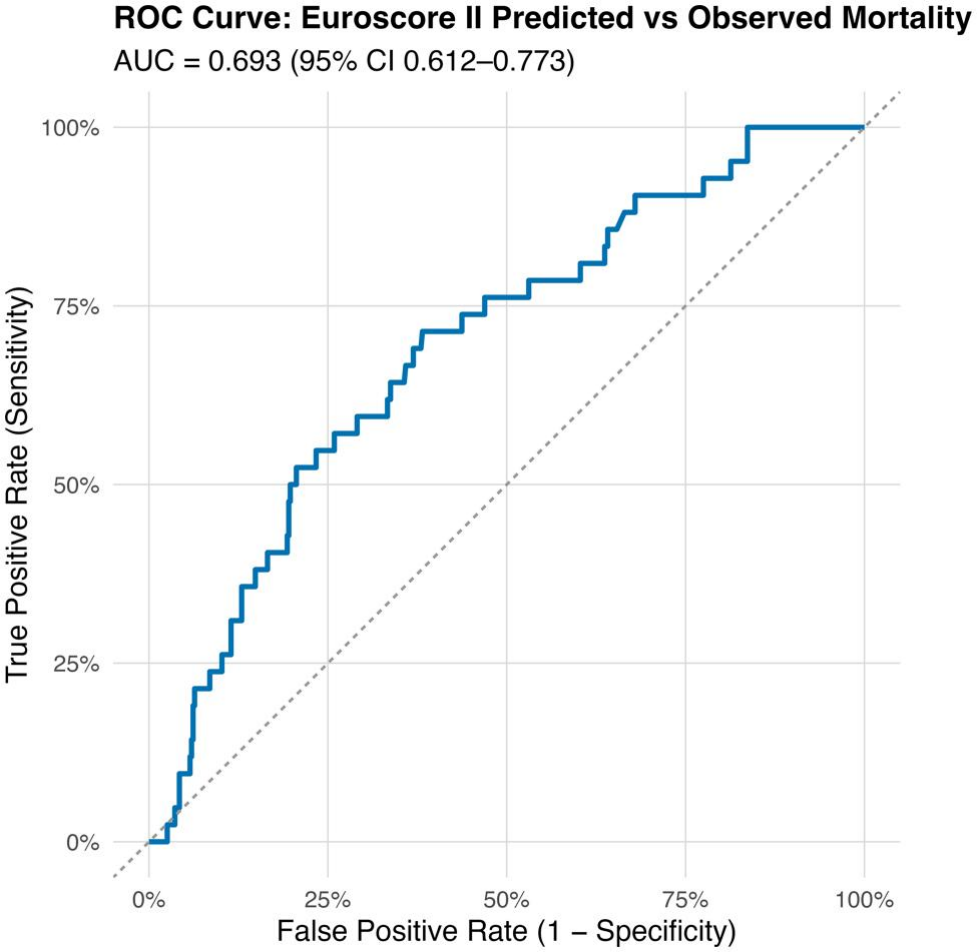
ROC Curve for the EuroSCORE 2 Model in the Dialysis Cohort. Abbreviations: AUC< area under the curve; ROC, receiver operating characteristic

The EuroSCORE II model demonstrated relatively poor discriminatory ability in the dialysis cohort, with an AUC of 0.693 (95% CI: 0.61-0.77; **Figure 5**).

#### Clinical utility

**Figure 6.**
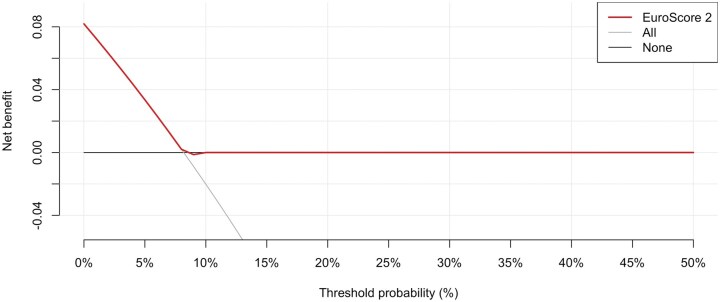
Decision-Curve Analysis for the EuroSCORE II (ES2) Model Predicting Postoperative Mortality in Dialysis Patients Undergoing CABG. The red line represents the ES2 model, while the grey and black lines indicate the “treat-all” and “treat-none” strategies, respectively

The decision-curve analysis (**Figure 6**) demonstrated that the EuroSCORE II model shows minimal clinical net benefit compared with the “treat-all” and “treat-none” strategies.

A slight advantage was observed only at very low risk probabilities (<8%).

## DISCUSSION

The findings of our study demonstrate significant in-hospital mortality and morbidity in patients on preoperative dialysis undergoing CABG. The propensity-matched analysis further strengthens the validity of the findings by controlling for confounders between the 2 study groups and by teasing out the impact of chronic dialysis on outcomes, since dialysis patients are a highly comorbid population. All previous studies were retrospective and did not use propensity-matching to account for this effect.

Despite the substantial early mortality of dialysis patients undergoing CABG, the use of the left internal mammary artery has been consistently associated with a reduced hazard of 5 years of long-term mortality.^16^ Furthermore, a post-hoc trial analysis of CKD patients enrolled in the SYNTAX trial at 5-year follow-up demonstrated a higher MACCE rate driven by repeat revascularization and all-cause death after PCI compared to CABG.^8^ Therefore, understanding the current outcomes of CABG in dialysis patients is critical to making the right decision for patients discussed by the Heart Team. The present study used a robust, real-world dataset from the NACSA in the UK to provide a comprehensive analysis of the impact of renal dialysis on surgical outcomes following CABG surgery. Therefore, the study adds valuable information for the decision-making of the Heart Team when faced with such challenging cases. Furthermore, to arrive at the optimal revascularization strategy for their patients, clinicians must also understand whether patients are suitable for future renal transplantation and weigh the risks and benefits of the intervention they offer in the context of a known significantly reduced survival of dialysis patients.^17^

These findings corroborate the existing and rather limited literature. Shroff et al conducted a retrospective analysis using United States Renal Data System data. They reported an in-hospital mortality rate of 8.2% among 6178 renal dialysis patients undergoing CABG surgery.^10^ Similarly, Cooper et al conducted a retrospective analysis using data from the Society of Thoracic Surgeons National Adult Cardiac Database to examine operative mortality in this population. Their study revealed a significantly higher operative mortality rate among 7152 dialysis-dependent patients compared to patients with normal renal function (9% vs 1.3%).^9^

Interestingly, despite the significant difference in mortality, the incidence of postoperative neurological complications did not differ significantly between the 2 groups. This suggests that while dialysis patients are at greater overall risk, their susceptibility to neurological complications may not be disproportionately elevated relative to other surgical risks. Our findings contrast with the study by Cooper et al, which found a more than 3-fold increase in stroke in patients on renal dialysis undergoing CABG compared to patients not on renal dialysis.^9^ As pointed out in the limitation section, the reporting of stroke outcomes (CVA and TIA) is not mandatory; hence, the lack of statistical difference could be due to low reported numbers.

Dialysis patients had a more extended median hospital stay of 12 days compared to 9 days in non-dialysis patients. This finding aligns with previous reports suggesting a longer recovery and increased health resource utilization for renal dialysis patients undergoing CABG.^18^ Moreover, dialysis patients had a higher risk of returning to theatre for bleeding, and this is likely related to a more profound coagulopathy that is more prevalent in this very high-risk cohort. This suggests again a significant impact on healthcare costs related to a higher theatre utilization and, most likely, a higher transfusion rate in this patient subgroup.

The decision between on-pump CABG and off-pump CABG as a strategy for surgical revascularisation may also impact the outcome. Although OPCAB is thought to mitigate the complications associated with cardiopulmonary bypass and aortic cross-clamping if an anaortic procedure is used, multiple randomized controlled trials showed no impact on postoperative acute kidney injury rates.^19^^,^^20^ One meta-analysis by Lim et al of retrospective studies showed comparable results between on-pump and off-pump CABG.^21^ Our study did not find off-pump CABG to be associated with a beneficial effect on in-hospital mortality since its use was not a significant predictor for mortality and was not retained in the final model.

While the use of arterial grafts was associated with beneficial effects in renal dialysis CABG patients,^10^ the use of a single, two, or more arterial grafts was not found to be a predictor for in-hospital mortality in our analysis. Our study lacks long-term survival, so assessing whether this difference would become more evident in the long term was not possible.

Our analysis identified key predictors of in-hospital mortality for the study population. Advanced age was a predictor of increased risk of in-hospital mortality in dialysis patients. Furthermore, other predictors include patients with preoperative NYHA class IV symptoms and pulmonary hypertension. This highlights the critical need for meticulous preoperative evaluation and optimisation in dialysis patients undergoing CABG. Emergency surgery was retained as a non-significant predictor in the backward regression model, likely due to the small sample size.

The prevalence of CABG in patients on chronic dialysis out of all CABG cases performed during the study period was low (0.2%). While the annual volume of dialysis cases fluctuated widely throughout the study, we have noted a steep increase after 2011, followed by a steady increase in numbers. This could be explained by a refinement in operative technique over the years and an increased experience and confidence in the preoperative and postoperative critical care management of these complex patients, which resulted in more dialysis patients being offered CABG.

Finally, our analysis shows that the EuroSCORE 2 is poorly calibrated for predicting in-hospital mortality in dialysis cases, underestimating mortality in higher-risk cases. Moreover, the EuroSCORE 2 model displayed suboptimal discrimination and limited clinical usefulness in the DCA. Therefore, the risk of adjustment for surgical benchmarking and clinical decision-making in MDTs cannot rely solely on EuroSCORE2, suggesting the development of improved prediction tools for this high-risk patient cohort.

### Limitations

While our study provides valuable insights, several limitations warrant discussion. The propensity-matched design inherently risks residual confounding despite our efforts to control this. Another limitation was the lack of long-term data follow-up to understand the survival of this unique patient population and the long-term impact of the type of arterial grafts. Another limitation was the lack of a PCI cohort to compare the results of CABG. Despite the rigorous audit process to ensure adequate data quality, some data parameters may be inaccurate due to the retrospective nature of the data, and some fields may not have been mandatory in the past, potentially resulting in reduced reporting because the study spans an extended period. This could be the case for post-op CVA or TIA rates. Furthermore, some of the trends we notice in the annual number of procedures could be genuine. Still, they might also be affected by improved reporting over the years, which is a limitation of all registry datasets.

## CONCLUSION

The study provided evidence of a steady overall increase in CABG operations performed on renal dialysis-dependent patients in the UK over the last 20 years. Preoperative chronic dialysis is associated with considerable in-hospital mortality, return to theatre for bleeding and prolonged recovery in patients undergoing CABG. The EuroSCORE2 mode showed poor calibration, discrimination, and minimal clinical benefit in predicting mortality in this patient cohort.

## Supplementary Material

ivaf291_Supplementary_Data

## Data Availability

The data underlying this study are available from the University of Bristol upon reasonable request and subject to a formal data-sharing agreement. Access will be granted to qualified researchers for the purpose of replicating or extending the analyses reported here. All shared data will be de-identified in accordance with data protection legislation, and recipients must agree not to attempt re-identification of participants. Data will be stored and transferred securely, in accordance with University of Bristol policies and GDPR requirements. Access requests should be directed to daniel.fudulu@bristol.ac.uk and will be reviewed by the study’s data governance committee to ensure compliance with ethical and legal standards.
