# Supplementary material for: Coronary Artery Bypass Surgery in Patients on Dialysis: In-Hospital Outcomes from UK Registry Analysis
Source: Interdiscip Cardiovasc Thorac Surg. 2025 Dec 3;40(12):ivaf291. doi: 10.1093/icvts/ivaf291 (PMC12782734; doi:10.1093/icvts/ivaf291)

**Supplementary file**

**Coronary artery by-pass surgery in patients on dialysis: in-hospital outcomes from UK registry analysis**

Muhammed A. Mashat^1,3^, Tim Dong1, Rahul Kota^1^, Ettorino Di Tommaso^1^, Pradeep Narayan^1,2^, Charles Tan^1^, Cha Rajakaruna^1^, Eltayeb Mohamed Ahmed^1^, Gianni D Angelini^1^, Daniel P Fudulu^1^

1. Bristol Heart Institute, University of Bristol
2. Department of Cardiac Surgery, Rabindranath Tagore International Institute of Cardiac Sciences, Narayana Health, Kolkata, India
3. King Abdulaziz University

Supplemental Figure 1 – Covariate overlap graph for baseline characteristics used during matching.


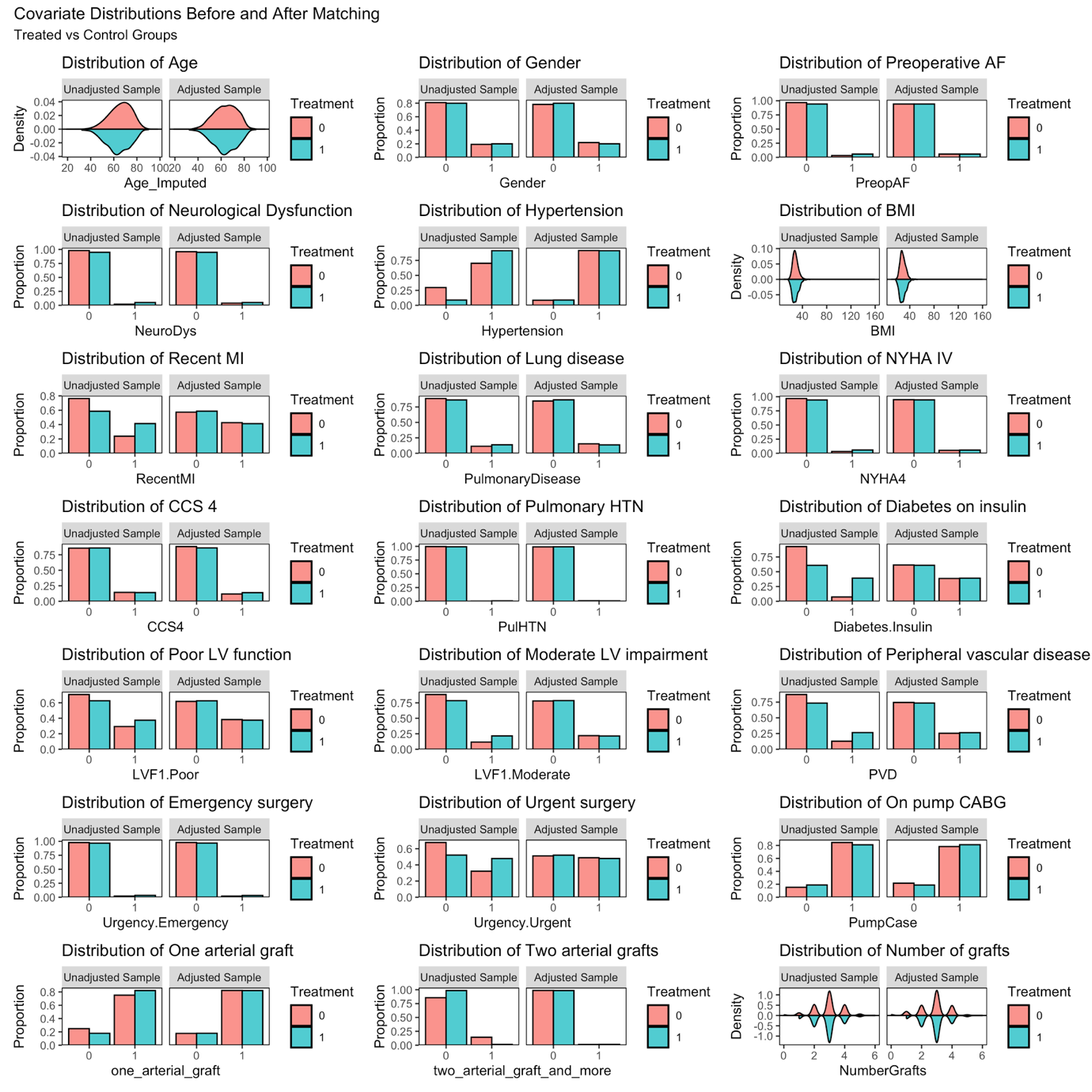

Supplement: ivaf291_Supplementary_Data [file ivaf291_supplementary_data.docx]
